# What Explains Usage of Mobile Physician-Rating Apps? Results From a Web-Based Questionnaire

**DOI:** 10.2196/jmir.3122

**Published:** 2014-06-11

**Authors:** Sonja Bidmon, Ralf Terlutter, Johanna Röttl

**Affiliations:** ^1^Department of Marketing and International ManagementAlpen-Adria Universitaet KlagenfurtKlagenfurt am WoertherseeAustria

**Keywords:** physician-rating apps, physician-rating websites, sociodemographic variables, psychographic variables, digital literacy, TAM

## Abstract

**Background:**

Consumers are increasingly accessing health-related information via mobile devices. Recently, several apps to rate and locate physicians have been released in the United States and Germany. However, knowledge about what kinds of variables explain usage of mobile physician-rating apps is still lacking.

**Objective:**

This study analyzes factors influencing the adoption of and willingness to pay for mobile physician-rating apps. A structural equation model was developed based on the Technology Acceptance Model and the literature on health-related information searches and usage of mobile apps. Relationships in the model were analyzed for moderating effects of physician-rating website (PRW) usage.

**Methods:**

A total of 1006 randomly selected German patients who had visited a general practitioner at least once in the 3 months before the beginning of the survey were randomly selected and surveyed. A total of 958 usable questionnaires were analyzed by partial least squares path modeling and moderator analyses.

**Results:**

The suggested model yielded a high model fit. We found that perceived ease of use (PEOU) of the Internet to gain health-related information, the sociodemographic variables age and gender, and the psychographic variables digital literacy, feelings about the Internet and other Web-based applications in general, patients’ value of health-related knowledgeability, as well as the information-seeking behavior variables regarding the amount of daily private Internet use for health-related information, frequency of using apps for health-related information in the past, and attitude toward PRWs significantly affected the adoption of mobile physician-rating apps. The sociodemographic variable age, but not gender, and the psychographic variables feelings about the Internet and other Web-based applications in general and patients’ value of health-related knowledgeability, but not digital literacy, were significant predictors of willingness to pay. Frequency of using apps for health-related information in the past and attitude toward PRWs, but not the amount of daily Internet use for health-related information, were significant predictors of willingness to pay. The perceived usefulness of the Internet to gain health-related information and the amount of daily Internet use in general did not have any significant effect on both of the endogenous variables. The moderation analysis with the group comparisons for users and nonusers of PRWs revealed that the attitude toward PRWs had significantly more impact on the adoption and willingness to pay for mobile physician-rating apps in the nonuser group.

**Conclusions:**

Important variables that contribute to the adoption of a mobile physician-rating app and the willingness to pay for it were identified. The results of this study are important for researchers because they can provide important insights about the variables that influence the acceptance of apps that allow for ratings of physicians. They are also useful for creators of mobile physician-rating apps because they can help tailor mobile physician-rating apps to the consumers’ characteristics and needs.

## Introduction

### Background

Technological advances have always had major impacts on medicine [1]. Many leading companies in the computer and Internet industries have entered the mobile marketplace, such as Google with the Android Mobile Operating System and Apple with the iPhone being the 2 dominant operating systems [2]. The smartphone is one of the fastest growing sectors in the technology industry and also has significant impact on medicine [1]. The number of people who use smartphones and mobile tablet computers is expanding rapidly. More than 95% of young US adults between the ages of 18 and 29 years own a mobile phone, and almost 30% of those use their mobile phone to look for health or medical information. In the United States, more than half of the adults aged 65 and older own a mobile phone [3]. Younger people are more likely than older people to own and use a smartphone. Regarding apps, recent studies show that there has been an enormous increase in the number of smartphone and tablet apps downloaded over the past years [4]. More than 300 million apps were downloaded in 2009 and more than 5 billion apps were downloaded in 2010 [5]. Patients use mobile devices and apps to manage and control their health, and 1 in 5 smartphone owners has at least 1 health app (eg, for diet, weight, and exercise) [5,6]. Hence, similar to developments in most consumer markets, consumers increasingly access health-related information via mobile devices. Mobile media devices are popular tools in the area of medicine because they allow for immediate information [7]. Smartphones and mobile tablet computers offer many advantages for patients in comparison to other technologies, such as mobility, capability, portability, intuitive and tactile graphical user interface, permanent connection to the Internet, and storage capacity [4,5,7-9].

An *app* is defined as “a software program for a computer or phone operating system” [9]. In this paper, we use the term *mobile apps* to describe “Internet applications that run on smartphones and other mobile devices” [9]. Apple offers the highest number of health-related apps of any platform. In 2010, Apple’s App Store offered more than 7136 health-related apps; 1296 health-related apps were offered by Google Android and 333 by BlackBerry [5]. In October 2013, the IMS Institute for Healthcare Informatics released a report on mobile health apps (all apps categorized as “health or wellness” or “medical” were reviewed) showing that 43,689 health care apps were currently available on the US iTunes Store [10]. The health application market is booming [11]. The Global Mobile Health Market Report estimates that by 2015 more than one-third of all 1.4 billion smartphone users will utilize a mobile health care app [11]. In Europe, Germany is one of the biggest app markets with average growth rates of 183% over the past 4 years [12].

Several recently released mobile apps allow consumers to rate and locate physicians , such as Vitals [13], Rate MDs [14], ZocDoc [15], and Healthgrades [16] in the United States, or Jameda [17], DocInsider [18], and Imedo [19] in Germany. However, there is practically no research on factors that contribute to the adoption of such mobile physician-rating apps.

Physician-rating websites (PRWs) provide patients with information on the quality of health care system participants, such as physicians or hospitals [20]. On the Internet, they are a source of peer-to-peer information about individual physicians [21], an opportunity to review a physician in an anonymous and self-driven way [22], and another way to find health information and make health-related decisions [23] in addition to the usually preferred sources of recommendation from friends, colleagues, and family members, or from other physicians (eg, finding a new general practitioner) [24]. The structure of PRWs is similar to the well-known rating systems on the Internet for travel websites, hotels, or restaurants [20]. There is an increasing number of PRWs throughout the world [25-27]. A controversial discussion about the utility and the impact of PRWs in several health systems has been ongoing [21]. A cross-sectional study by Emmert et al in 2013 [28] showed that approximately one-third of an online sample in Germany was aware of the existence of German PRWs and approximately one-quarter had searched for a physician on a PRW at least once in the past. Compared to a study in the United States conducted in 2010 [29] in which 16% of Internet users and 19% of people who were looking for health-related information on the Internet had used a PRW, a slight increase of usage can be seen, but usage is still at a relatively low level. According to Emmert et al [28], people who have already posted a rating on a German PRW belong to the minority. Poor usage goes hand in hand with a small number of patient satisfaction/experience ratings per physician [20,30,31]. A study conducted by Terlutter et al in 2012 [32] found that younger, male, more highly educated people and those people with a chronic disease were more inclined to use PRWs. Users also differed psychographically from nonusers of PRWs because they revealed more positive feelings about the Internet and other Web-based applications in general and had a higher digital literacy rate than nonusers. Users ascribed higher usefulness to PRWs than nonusers, trusted information on PRWs to a greater degree, and were more likely to rate a physician on a PRW in the future as well as to use them in the future [32]. The study further showed that sociodemographic variables and health status alone did not satisfactorily predict usage or nonusage of PRWs, but that psychographic variables and variables of information-seeking behavior were needed to predict usage of PRWs [32].

Applying PRWs through mobile apps could be a way to boost usage of PRWs in general. A mobile physician-rating app transfers existing functionality of PRWs to the mobile realm, possibly making it easier and more flexible for patients to both access information and provide information (rate a physician). Consequently, this paper aims at delivering important insights into the usage of mobile physician-rating apps by looking at what kind of variables explain adoption of mobile physician-rating apps. With this knowledge, creators of mobile physician-rating apps could better tailor them to the consumers’ characteristics and needs.

Because some of the apps are available free of charge, whereas others are only available at a cost (typically a relatively small fee), we are also interested in patients’ willingness to pay for mobile physician-rating apps.

### Conceptual Model

**Figure 1:**
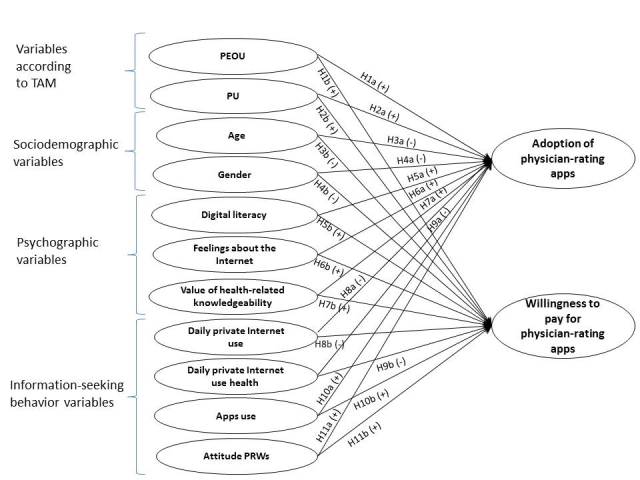
Theoretical model of adoption of physician-rating (PR) apps and willingness to pay for them showing various hypothesized (H) relationships. A plus or minus sign signifies an increase or decrease, respectively, in the dependent variable evoked by an increase in the independent variable (ceteris paribus). PEOU: perceived ease of use; PRW: physician-rating website; PU: perceived usefulness; TAM: Technology Acceptance Model.

This study proposes a causal model consisting of different antecedents of adoption of mobile physician-rating apps and willingness to pay for them (Figure 1). A plus sign or minus sign signifies an increase or decrease, respectively, in the dependent variable evoked by an increase in the independent variable (ceteris paribus). The relationships and expected directions of influence are described in detail subsequently.

### Technology Acceptance Model

The Technology Acceptance Model (TAM) [33,34], based on the Theory of Reasoned Action, is an applied and widely used model to describe and predict the acceptance and use of new information technology. The model focuses on what attributes of a certain technology increase the acceptance of a technology. According to TAM, acceptance of a technology depends on how the technology is perceived. TAM identified perceived usefulness and perceived ease of use (PEOU) as 2 central beliefs about a new technology which influence the attitude toward and the use of that technology [35-38]. The perceived usefulness is defined as “the user’s perception of the degree to which using a particular system will improve her/his performance” [38]. The PEOU is defined as the “user’s perception of the extent to which using a particular system will be free of effort” [38]. The TAM has been supported by many studies and has been applied in different contexts of online consumer behavior [39,40], including the area of health information websites [41] or mobile health services [42]. According to Kim and Chang [41], perceived usefulness and PEOU are “key factors in accepting information technology like health information service on the Internet.” Given the broad support of PEOU and perceived usefulness for understanding acceptance of new technologies, the 2 constructs from TAM have been included in our model. In our study, we conceptualize perceived usefulness as the usefulness of the Internet to gain health-related information and PEOU as the perceived ease of use of the Internet to gain health-related information, and we expect both variables to have a significant impact on the adoption and willingness to pay for a mobile physician-rating app. It is hypothesized that:

H1a: Individuals ascribing higher ease of use (PEOU) to the Internet to gain health-related information are more likely to adopt a mobile physician-rating app.

H1b: Individuals ascribing higher ease of use (PEOU) to the Internet to gain health-related information are more willing to pay for a mobile physician-rating app.

H2a: Individuals ascribing higher usefulness to the Internet to gain health-related information are more likely to adopt a mobile physician-rating app.

H2b: Individuals ascribing higher usefulness to the Internet to gain health-related information are more likely to pay for a mobile physician-rating app.

In addition, we extended the model with variables related to health information search that were identified in an extensive literature review. Sociodemographic variables (eg, age, gender), psychographic variables (eg, digital literacy, feelings about the Internet and other Web-based applications in general, patients’ value of health-related knowledgeability), and information-seeking behavior variables (eg, daily private Internet use and daily private Internet use for health-related information, frequency of using apps for health-related information in the past, attitude toward PRWs) were included in the final model.

### Sociodemographic Variables

#### Age

Age is likely to be an important predictor of evaluation of and behavior toward mobile physician-rating apps. Similar to the use of the Internet in general [3,43-49], mobile Internet use also declines with increasing age. The German Digitalbarometer I/2012 reported that 44% of people in the age range of 14-29 years, 28% in the age range of 30-49 years, and 10% of people older than 50 years used apps [50]. In 2013, another German online study showed that the use of apps decreases continuously with rising age: 70% of people aged between 14 and 29 years used apps, 46% aged between 30 and 49 years, 24% aged between 50 and 69 years, and only 12% age 70 years and older [51]. Charness and Boot [52] identified attitudinal barriers, cognitive barriers (eg, fluid and crystallized intelligence, computer anxiety), as well as age-related changes (eg, in perceptual, cognitive, and motor systems) affecting technology use and greater privacy concerns that lead to the lag of older adults in technology adoption. Therefore, it can be suggested that:

H3a: Younger people are more likely to adopt a mobile physician-rating app than older people.

H3b: Younger people are more willing to pay for a mobile physician-rating app than older people.

#### Gender

Even though women are typically more inclined to use the Internet for health-related information [2,53-59], when it comes to mobile usage of the Internet, men are more likely to use the Internet on their mobile phone than women, as reported in an European eHealth survey by Kummervold et al [60], for example. According to the German ARD/ZDF online study in 2013, 46% of men, but only 36% of women, were mobile users of the Internet [51]. Another German study in 2013 revealed that 58.7% of mobile Internet users were male and 41.3% were female [61]. The German Digitalbarometer I/2012 reported that 36% of men and 18% of women used apps [50]. One explanation for the higher usage of mobile devices and apps by men than by women may be lower levels of computer anxiety and higher perceived behavioral control by men than by women [62]. In summary, men consistently show higher levels of mobile Internet and app usage than women do. Therefore, we hypothesize that:

H4a: Men are more likely to adopt a mobile physician-rating app than women are.

H4b: Men are more likely to pay for a mobile physician-rating app than women are.

### Psychographic Variables

#### Digital Literacy

Digital literacy describes the ability to effectively and critically use a range of digital technologies. High levels of digital literacy enable individuals to make responsible choices and to access information and ideas in the digital world and share them with others and it is deemed an important prerequisite in today’s digital world [63]. High levels of literacy in the digital domain are seen as leading to many social and psychological benefits across the life span [64]. However, low levels of literacy can pose barriers to the access and use of health information and eHealth tools, especially if paired with low health literacy [65]. A digitally literate individual is able to make use of different technical devices and use these to his or her advantage. Therefore, we expect that a higher level of digital literacy likely leads to a higher affinity for new digital offers, especially when designed to facilitate the use of digital content, such as an app. In addition, it has been demonstrated that people with higher digital literacy show less computer anxiety [66], which also likely leads to greater openness toward new offers. Therefore, the following hypotheses can be assumed:

H5a: Individuals with a higher digital literacy are more likely to adopt a mobile physician-rating app.

H5b: Individuals with a higher digital literacy are more willing to pay for a mobile physician-rating app.

#### Feelings About the Internet and Other Web-Based Applications

Whereas digital literacy primarily concerns the ability to make use of digital technologies and information, people also hold more or less positive or negative affective evaluations or feelings toward the Internet or other Web-based applications [39,67]. If they hold more favorable feelings, they are more likely willing to adopt new technologies. Thus, it can be suggested that:

H6a: Individuals with more positive feelings about the Internet and other Web-based applications in general are more likely to adopt a mobile physician-rating app.

H6b: Individuals with more positive feelings about the Internet and other Web-based applications in general are more willing to pay for a mobile physician-rating app.

#### Patients’ Value of Health-Related Knowledgeability

Literature has shown that the amount of information a person is seeking and the amount of cognitive effort and elaboration a person is willing to devote to a specific task may vary substantially based on the personality of the individual [40,68]. Whereas some patients are inclined to prepare themselves for visiting a doctor and search for health-related information, others search for health-related information to a lesser extent. Patients who value health-related knowledgeability more highly (eg, believe being well informed leads to better patient-physician communication or that the physician offers more time to well-informed patients) are inclined to make significant health decisions on the basis of health-related information found on the Internet [39,68]. They even decide whether professional medical care is needed; alternatively, they decide whether to rely on self-treatment based on their online findings [69]. Patients with a high value of health-related knowledgeability are used to searching for health-related information on the Internet to a greater extent than individuals who have a lower value of health-related knowledgeability. Therefore, these patients may evaluate a mobile physician-rating app to be a useful amendment to a health-related information search. This leads us to the following hypothesis:

H7a: Patients with a higher value of health-related knowledgeability are more likely to adopt a mobile physician-rating app.

H7b: Patients with a higher value of health-related knowledgeability are more willing to pay for a mobile physician-rating app.

### Information-Seeking Behavior Variables

#### Daily Private Internet Use and Use for Health-Related Information

According to a recent study conducted in Germany, 1 of the 2 main motivations for people to own a tablet personal computer or a smartphone that enables mobile access to the Internet is saving time [70]. Between 2011 and 2013, the number of respondents who used mobile Internet over their smartphone or mobile phone because they wanted to save time rose from 51.9% to 57.6% [71]. Because mobile access allows for fast and flexible access to the Internet, it can be assumed that people who have a strong motivation to save time spend less time on private Internet use in general and also on searching for health-related information on the Internet. In turn, we expect that people who spend less time on private Internet use in general and on searching for health-related information should be more interested in a physician-rating app because they may be under more time pressure and may be looking for fast alternatives to a health-related information search. This leads us to the following hypotheses:

H8a: Individuals with a higher amount of daily private Internet use in general are less likely to adopt a mobile physician-rating app.

H8b: Individuals with higher amount of daily private Internet use in general are less willing to pay for a mobile physician-rating app.

H9a: Individuals with a higher amount of daily private Internet use for health-related information search are less likely to adopt a mobile physician-rating app.

H9b: Individuals with higher amount of daily private Internet use for health-related information search are less likely to pay for a mobile physician-rating app.

#### Past Use of Apps for Health-Related Information

Patients can make use of different devices to search the Internet for health-related information, the most prominent being personal computer, laptop, smartphone, or mobile tablet computer. Apps are designed primarily for use with smartphones or mobile tablet computers, and along with the massive expansion of these mobile devices, usage of apps for different purposes has increased significantly. According to a study conducted in Germany in November 2012, there were 43.7 apps on average installed on an iPhone, 28 apps on an Android Smartphone, 32.9 apps on an iPad, and 36.1 apps on an Android Tablet [72], including apps for health and fitness issues. More than half of the apps installed were actually used by the consumer [72]. A systematic review investigating patient acceptance of consumer health information technology found out that prior experience or exposure to computer and/or health technology increases its acceptance [62]. Of the 20 studies investigating the effects of different dimensions of prior experience to computer/health technology, 15 confirmed that prior experience was associated with increased acceptance [62]. We can assume that individuals who already make use of health-related apps more frequently are probably more open toward a mobile physician-rating app. So we conclude from the usage of health-related apps to the likely usage of a mobile physician-rating app:

H10a: Individuals who use apps more frequently for health-related information in the past are more likely to adopt a mobile physician-rating app.

H10b: Individuals who use apps more frequently for health-related information in the past are more willing to pay for a mobile physician-rating app.

#### Attitude Toward Physican Rating Websites

We also assume a positive influence of the patients’ attitude toward PRWs in general on patients’ perception of physician-rating apps. If patients’ overall attitude toward PRWs is positive, patients are likely to be more positive toward apps that facilitate access to the PRW. This leads us to the final hypotheses:

H11a: Individuals who have a better attitude toward PRWs are more likely to adopt a mobile physician-rating app.

H11b: Individuals who have a better attitude toward PRWs are more willing to pay for a mobile physician-rating app.

#### Moderator Analysis: Users vs Nonusers of Physician-Rating Websites

It might be expected that respondents who have already used PRWs on some technological (nonmobile) device in the past behave differently with regards to the adoption of mobile physician-rating apps than those who have no experience. Hence, we explore whether the usage of PRWs moderates the relationships in the conceptual model. According to Baron and Kenney [73], a moderator is a “qualitative (eg, sex, race, class) or quantitative (eg, level of reward) variable that affects the direction and/or strength of the relation between the independent or predictor variable and a dependent or criterion variable.”

## Methods

### Participant Recruitment

An online survey of 1006 German patients was conducted in September 2012. The sample was drawn from an e-panel maintained by GfK HealthCare, a leading survey research company in Nuremberg, Germany. It was based on a randomly generated set of users who had visited a general practitioner at least once in the 3 months before the beginning of the survey. In all, 1561 people were contacted; 555 people could not participate because they had not visited a general practitioner within the past 3 months. The recruitment rate was 64.4% [74]. Another 20 participants were excluded from the analysis because of an extremely short response time and inconsistent answer patterns (eg, flatliners, contradictions). Another 28 respondents were excluded because their number of missing values exceeded the limit of 30% [75] in scale items. The final sample consisted of 958 participants. Small monetary incentives were offered for survey completion.

### Questionnaire

The survey was designed by the researchers based on the existing literature. All items (except categorizing variables) were measured with 7-point rating scales. For construct measures used in the final partial least squares (PLS) model and sociodemographic measures see Multimedia Appendix 1. Existing scales from the literature were used where applicable. The data were checked and missing values were imputed with SPSS version 20 (IBM Corp, Armonk, NY, USA). The data were analyzed by PLS path modeling with the software SmartPLS.

### Measurement Model

#### Overview

The PEOU and perceived usefulness of the Internet to gain health-related information were measured by existing multi-item scales derived and adapted from Venkatesh and Davis [36]; PEOU was entered into SmartPLS with 2 items, PU was entered with 3 items. Age and gender were measured by a single item (year of birth and gender, respectively). Digital literacy was measured with an item based on Norman and Skinner [76] (1=not literate at all, 7=very literate). Feelings about the Internet and other Web-based applications in general were measured by an item derived from Porter and Donthu [35] (1=very negative, 7=very positive). Patients’ value of health-related knowledgeability was measured with a scale of 9 items, which was developed by the researchers. Some items were adapted from the health information orientation scale by Dutta-Bergman [77]. Exploratory factor analysis revealed a single factor solution, explaining 53.88% of variance. Factor loadings ranged from .639 to .807; Cronbach alpha was .892. Items were reduced for modeling. The 3 items with the highest outer weights were included in the final model. Total daily private Internet use in general and total daily Internet use for health-related information searches were measured in hours per day (or alternatively per week or per month) with 2 separate questions. Measures were subsequently recoded into the average measure of hours per day in general and hours per day for health-related information searches. The frequency of using apps for health-related information in the past was measured with the item “How often do you use apps for health-related information searching on the Internet?” (1=daily, 2=weekly, 3=less often than weekly, 4=monthly, 5=less often than monthly, 6=never). This variable was coded inversely; therefore, the variable was recoded before entering the SmartPLS model. Attitude toward PRWs was measured by 3 items representing trust in PRWs, utility of PRWs, and intention to use them in the future. All these questions had a “no answer” category as an alternative.

#### Moderator Variable: Usage vs Nonusage of PRWs

The moderator variable “experience with PRWs” was measured dichotomously with the following wording: “Have you ever gathered information about a physician on a physician-rating site?” (1=yes, 2=no, 3=no answer). A total of 15 respondents chose the no answer category and were excluded from the subsequent group comparisons.

#### Endogenous Variables: Adoption of Physician-Rating Apps and Willingness to Pay for Them

Respondents were asked to think of a mobile physician-rating app and decide how much they would appreciate it and how much they would pay for it. We asked respondents to imagine a physician-rating app for several reasons. First, as outlined by Emmert et al [28], PRW use is relatively low in general and usage would be even lower when we focused on mobile usage. Secondly, we wanted to avoid asking participants about a specific physician-rating app only because such apps differ in their quality and distribution and are not yet widespread. By describing a physician-rating app and asking participants to imagine it, we were able to realize a substantial number of evaluations and could base them on comparable stimuli. The following text was used as introduction: “Imagine that there exists an app for smartphones to search for physicians. The user could fill in a symptom of a condition and as a result all physicians in the surrounding area were listed, including the ratings of these physicians according to the satisfaction of the rating patients with him/her, with the atmosphere of the waiting room, waiting time, the treatment, et cetera.“

Adoption of physician-rating apps was measured by asking respondents to indicate their agreement with the following 2 items on a 7-point rating scale (1=strongly disagree; 7=strongly agree): (1) I appreciate such an app, and (2) I am willing to use such an app.

Willingness to pay for physician-rating apps was measured by the item “I am willing to pay for such an app.” Again, participants could indicate their agreement on a 7-point rating scale (1=strongly disagree; 7=strongly agree).

### Analytical Procedure

The causal relationships between the constructs were analyzed through structural equation modeling using the PLS approach, as implemented in the free software environment of SmartPLS [78]. PLS has found prevalent usage in the area of technology adoption and information systems literature [79], particularly because it is also well suited for research in its early stages when the focus is on saturated prediction-oriented models [80]. Bootstrapping with 5000 bootstrap samples to receive inference statistics was applied. To assess PLS path models, the results were evaluated in a 2-step process. First, the measurement models were analyzed, evaluating the reliability and validity of the estimates for the latent variables. Second, the structural (inner) model was assessed [81,82].

We calculated a PLS analysis for the total sample (N=958) and in a second step for the 2 groups of users (n=254) and nonusers (n=689) of PRWs. The quality of the fit of the measurement model was evaluated extensively and was based on the criteria formulated by Ringle et al [79]. Fit measures were calculated for the total sample and for both subsamples. Factor loadings, composite reliability, and average variance extracted were used to evaluate local fit of the constructs. The internal consistency reliability was evaluated using Cronbach alpha. Convergent validity was evaluated based on the average variance extracted. For assessing discriminant validity, the Fornell-Larcker criterion was applied [83]. Finally, multicollinearity was checked.

## Results

### Sample Characteristics

A comparison of the sample of the current study and the 2012 German Internet users (the German online population) [84] reveals that the sample represents the German online population quite well concerning the sociodemographic variables (Table 1). With regard to gender, the sample mirrors the German online population well. Regarding age, participants in our sample were slightly older than those in the German online population. The reason for this deviation lies probably in the selection criterion for participation; to qualify for our study, participants must have visited a general practitioner at least once in the previous 3 months. With regard to education, the percentage of respondents with higher education was larger in our sample than in the German online population. There were no comparable data in the German online population regarding marital status or household size.

**Table 1:** Overview of study sample in comparison with German Internet population (2012).

Variable and category		Study sample data N=958	German Internet users (rounded to 1000 people) N=57,045,000
**Gender, n (%)**			
	Men	517 (54.0)	29,553,000 (51.8)
	Women	441 (46.0)	27,492,000 (48.2)
**Age (years), mean (SD)**		43.73 (13.0)	
	Age limits (years)	18-70	>10
**Age dichotomized, n (%)**			
	<44 years	471 (49.2)	32,896,000 (57.7)
	45-70 years	487 (50.8)	24,147,000 (42.3)
**Age categories (years), n (%)**			
	<24	81 (8.5)	12,552,000 (22.0)
	25-44	390 (40.7)	20,344,000 (35.6)
	45-64	431 (45.0)	18,799,000 (33.0)
	>65	56 (5.8)	5,348,000 (9.4)
**Education, n (%)**		951 (100.0)	52,589,000 (100.0)
	Without school qualification	4 (0.4)	Low education: 9,487,000 (18.0)
	Secondary general school	13 (1.4)	
	Polytechnic secondary school	120 (12.5)	Medium education: 29,467,000 (56.0)
	Intermediate secondary school	269 (28.1)	
	Matura examination or higher	545 (57.0)	High education: 13,635,000 (26.0)
**Household, n (%)**		956 (100)	
	1	207 (21.6)	
	2	363 (37.9)	
	3-4	355 (37.1)	
	>4	31 (3.2)	
**Marital status, n (%)**		948 (100.0)	
	Single	200 (20.9)	
	Close-partnered	215 (22.4)	
	Married	460 (48.0)	
	Divorced	64 (6.7)	
	Widowed	9 (0.9)	

### Evaluation of the Measurement Model

The measurement models yielded adequate fit for the total sample and for users of PRWs and nonusers of PRWs groups (Tables 2 and 3). None of the local fit indicators of the measurement models, such as factor loading, composite reliability (CR), average variance extracted (AVE), were violated and values of Cronbach alpha were relatively high.

The Fornell-Larcker criterion [83] revealed that discriminant validity of the constructs is also supported. Each given construct is clearly different from the measures of other constructs [85]. The square roots of AVE values were all well above the values in the appropriate rows and columns of the correlation matrix of latent variables (Table 4). Further, cross loadings show that all items had the highest loadings on their respective construct and every construct loaded highest with its own items. Discriminant validity was also supported for the 2 subsamples.

**Table 2:** Fit of measurement model including factor loading, composite reliability (CR), average variance extracted (AVE), and Cronbach alpha of endogenous constructs for the final model in the total sample (N=958).

Composite and item^a^		Mean (SD)	Loading	AVE	CR	Cronbach alpha
**PEOU**						
	F11_1	6.26 (1.16)	0.95	0.90	0.95	.89
	F11_2	6.14 (1.17)	0.95			
**PU**						
	F11_11	4.37 (1.97)	0.85	0.74	0.89	.82
	F11_12	4.10 (1.98)	0.88			
	F11_15	3.85 (1.94)	0.85			
Age (S2_1)		43.73 (13.04)	1.00	—	—	—
Gender (D1)		—	1.00	—	—	—
Digital literacy (F2_1)		5.87 (1.06)	1.00	—	—	—
Feelings about the Internet (F1_1)		5.78 (1.11)	1.00	—	—	—
**Value of health-related knowledgeability**						
	F20_5	4.71 (1.71)	0.78	0.74	0.89	.82
	F20_8	3.37 (1.89)	0.89			
	F20_9	3.61 (1.93)	0.90			
Daily private Internet use (F3_per day)		3.10 (2.29)	1.00	—	—	—
Daily private Internet use health (F4_per day)		0.43 (1.53)	1.00	—	—	—
Apps use (F7_10recoded)		—	1.00	—	—	—
**Attitude toward PRWs**						
	F25_1	4.18 (2.00)	0.93	0.87	0.95	.93
	F26_1	4.27 (1.92)	0.94			
	F27_1	3.59 (1.63)	0.94			

^a^ PEOU: perceived ease of use; PU: perceived usefulness. The denomination of measurement variables corresponds to the denomination of the items in Multimedia Appendix 1.

**Table 3:** Fit of measurement model including factor loading, composite reliability (CR), average variance extracted (AVE), and Cronbach alpha of endogenous constructs for the final model in the subsample of users of PRWs (n=254) and the subsample of nonusers of PRWs (n=689).

Composite and item^a^		Mean (SD)		Loading		AVE		CR		Cronbach alpha	
		Users	Nonusers	Users	Nonusers	Users	Nonusers	Users	Nonusers	Users	Nonusers
**PEOU**											
	F11_1	6.45 (0.95)	6.21 (1.20)	0.94	0.95	0.90	0.90	0.95	0.95	.90	.89
	F11_2	6.36 (0.91)	6.07 (1.23)	0.96	0.95						
**PU**											
	F11_11	4.48 (1.88)	4.32 (2.01)	0.81	0.86	0.71	0.75	0.88	0.90	.80	.83
	F11_12	4.21 (1.96)	4.06 (1.99)	0.87	0.88						
	F11_15	4.03 (1.85)	3.78 (1.98)	0.85	0.84						
Age (S2_1)		42.39 (12.92)	44.37 (13.00)	1.00	1.00	—	—	—	—	—	—
Gender (D1)		—	—	1.00	1.00	—	—	—	—	—	—
Digital literacy (F2_1)		6.09 (0.95)	5.78 (1.09)	1.00	1.00	—	—	—	—	—	—
Feelings about the Internet (F1_1)		5.97 (1.01)	5.73 (1.12)	1.00	1.00	—	—	—	—	—	—
**Value of health-related knowledgeability**											
	F20_5	5.07 (1.57)	4.58 (1.74)	0.72	0.79	0.71	0.74	0.88	0.90	.80	.82
	F20_8	3.76 (1.87)	3.21 (1.88)	0.90	0.88						
	F20_9	4.01 (1.93)	3.45 (1.92)	0.89	0.90						
Daily private Internet use (F3_per day)		3.17 (2.04)	3.05 (2.36)	1.00	1.00	—	—	—	—	—	—
Daily private Internet use health (F4_per day)		0.55 (1.78)	0.39 (1.44)	1.00	1.00	—	—	—	—	—	—
Apps use (F7_10recoded)		—	—	1.00	1.00	—	—	—	—	—	—
**Attitude toward PRWs**											
	F25_1	5.47 (1.44)	3.71 (1.98)	0.85	0.93	0.78	0.88	0.91	0.96	.86	.93
	F26_1	5.24 (1.45)	3.91 (1.95)	0.91	0.94						
	F27_1	4.43 (1.32)	3.28 (1.64)	0.90	0.94						

^a^ PEOU: perceived ease of use; PU: perceived usefulness. The denomination of measurement variables corresponds to the denomination of the items in Multimedia Appendix 1.

**Table 4:** Correlation matrix of the latent constructs with square root of average variance extracted (AVE) in the diagonal (total sample).

Construct^a^		1	2	3	4	5	6	7	8	9	10	11	12	13
1	PEOU	0.94												
2	PU	0.22	0.86											
3	Age	0.07	–0.09	—										
4	Gender	0.06	0.04	–0.18	—									
5	Digital literacy	0.19	0.17	–0.15	–0.13	—								
6	Feelings about the Internet	0.25	0.19	–0.11	–0.02	0.49	—							
7	Value of health-related knowledgeability	0.14	0.32	0.01	0.01	0.20	0.16	0.86						
8	Daily private Internet use	–0.01	0.20	–0.19	0.03	0.18	0.18	0.16	—					
9	Daily private Internet use health	–0.07	0.09	–0.05	0.06	0.03	0.05	0.09	0.21	—				
10	Apps use	0.29	–0.11	–0.27	–0.05	0.21	0.24	0.23	0.15	0.17	—			
11	Attitude toward PRWs	0.26	0.34	–0.07	0.06	0.18	0.20	0.46	0.10	0.16	0.19	0.93		
12	Adoption of physician-rating apps	0.23	0.28	–0.17	–0.04	0.30	0.36	0.34	0.13	0.03	0.31	0.53	—	
13	Willingness to pay for physician-rating apps	–0.01	0.24	–0.16	0.03	0.16	0.20	0.33	0.15	0.13	0.37	0.39	0.53	0.97

^a^ PEOU: perceived ease of use; PU: perceived usefulness.

### Evaluation of the Structural Model

**Figure 2:**
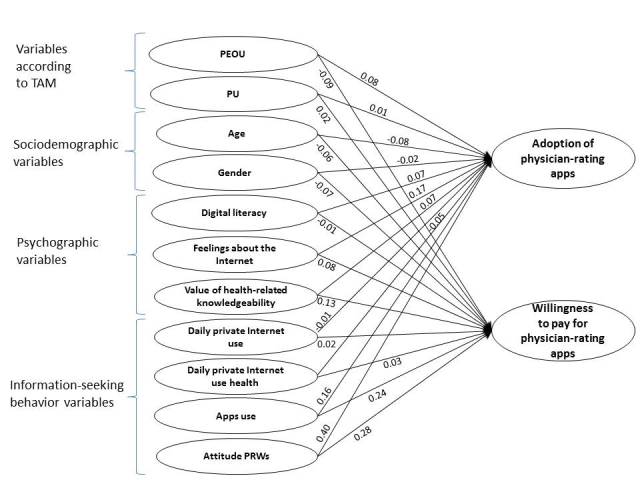
Structural model for the total sample. PEOU: perceived ease of use; PRW: physician-rating website; PU: perceived usefulness; TAM: Technology Acceptance Model.

Given that the measurement model yielded an acceptable fit, the structural model could be evaluated. The factors included in the conceptual model explained 40% of variance for adoption of physician-rating apps (*R* ^*2*^=.40) and 28% for willingness to pay for physician-rating apps (*R* ^*2*^=.28). Bootstrapping with 5000 samples revealed that 14 of 22 path coefficients of the conceptual model were significant. Figure 2 shows the results for model estimation of the total sample.

### Evaluation of the Hypotheses

Hypotheses testing results are reported in Table 5. As can be seen from the standardized beta coefficients (B), the hypotheses H1a, H3a + b, H4a, H5a, H6a + b, H7a + b, H9a, H10a + b, H11a + b are supported. The results for the total sample reveals that the present attitude toward PRWs is the most important factor to predict adoption of physician-rating apps and willingness to pay for a physician-rating app. In addition, the frequency of the use of apps for health-related information in the past also predicts adoption of physician-rating apps and willingness to pay to a high degree. Feelings toward the Internet and other Web-based applications also have a significant influence on adoption of physician-rating apps and they also predict willingness to pay for physician-rating apps. The patients’ value of health-related knowledgeability has a significant impact on adoption of physician-rating apps and an even stronger influence on willingness to pay for physician-rating apps. There is a significant positive influence of PEOU of the Internet to gain health-related information on adoption of mobile physician-rating apps and, in contrast to our initial predictions, a significantly negative influence on willingness to pay for physician-rating apps. As expected, age influences adoption and willingness to pay in a negative way (increasing age impedes willingness to adopt and to pay for physician-rating apps). Digital literacy has a significant, although weak, impact on the adoption of mobile physician-rating apps and no impact on willingness to pay. The daily Internet use for health-related information searches has significant impact on adoption of physician-rating apps, but not on willingness to pay for them. Gender influences adoption of a mobile physician-rating app and appears to affect willingness to pay for it although this was nonsignificant. As was expected, men were more prone to adopt physician-rating apps. In contrast to our initial predictions, perceived usefulness of the Internet to gain health-related information and the amount of daily private Internet use in general did not exert significant influence on the endogenous variables.

**Table 5:** Summary of partial least squares (PLS) estimation from the total sample (N=958).

Hypothesis	Path^a^	B	*t*_941_	*P* (1-sided)	Hypothesis testing results
H1a	PEOU → adoption (+)	0.08	3.26	.001	Supported
H1b	PEOU → willingness to pay (+)	–0.08	2.72	.003	Rejected
H2a	PU → adoption (+)	0.01	0.33	.37	Rejected
H2b	PU → willingness to pay (+)	0.03	0.83	.30	Rejected
H3a	Age → adoption (-)	–0.10	3.48	<.001	Supported
H3b	Age → willingness to pay (-)	–0.07	2.33	.01	Supported
H4a	Gender → adoption (-)	–0.07	2.58	.01	Supported
H4b	Gender → willingness to pay (-)	–0.05	1.59	.06	Rejected
H5a	Digital literacy → adoption (+)	0.05	1.79	.04	Supported
H5b	Digital literacy → willingness to pay (+)	–0.02	0.50	.31	Rejected
H6a	Feelings → adoption (+)	0.18	5.69	<.001	Supported
H6b	Feelings → willingness to pay (+)	0.08	2.41	.01	Supported
H7a	Patients’ value of health-related knowledgeability → adoption (+)	0.07	2.09	.02	Supported
H7b	Patients’ value of health-related knowledgeability → willingness to pay (+)	0.13	3.70	<.001	Supported
H8a	Internet use → adoption (-)	0.01	0.49	.31	Rejected
H8b	Internet use → willingness to pay (-)	0.02	0.50	.31	Rejected
H9a	Internet use health → adoption (-)	–0.04	1.99	.02	Supported
H9b	Internet use health → willingness to pay (-)	0.03	1.10	.14	Rejected
H10a	Apps use → adoption (+)	0.15	5.19	<.001	Supported
H10b	Apps use → willingness to pay (+)	0.23	6.16	<.001	Supported
H11a	Attitude PRWs → adoption (+)	0.41	12.57	<.001	Supported
H11b	Attitude PRWs → willingness to pay (+)	0.28	7.77	<.001	Supported

^a^ PEOU: perceived ease of use; PU: perceived usefulness. A plus sign or minus sign signifies an increase or decrease, respectively, in the dependent variable evoked by an increase in the independent variable (ceteris paribus).

### Group Comparisons: Usage vs Nonusage of Physician-Rating Websites

As outlined previously, a moderation analysis of usage of PRWs was carried out and the structural model was estimated for the 2 groups of users and nonusers of PRWs to explore whether the relationships in the model varied depending on the moderator variable. The model for users of PRWs explained 33% of variance for adoption of physician-rating apps (*R* ^*2*^=.33) and 27% for willingness to pay for physician-rating apps (*R* ^*2*^=.27), and the model for nonusers of PRWs explained 42% of variance for adoption of physician-rating apps (*R* ^*2*^=.42) and 28% for willingness to pay for physician-rating apps (*R* ^*2*^=.28).

A permutation test [86] was applied to examine the significance of group differences in path coefficients between the 2 subsamples. The path differences were tested running 1000 permutation samples for each model comparison [87]. The results can be seen in Table 6. The *P* value indicates the percentage of how many sampled path differences are greater or less than the observed path differences (2-sided test). The group comparison between users and nonusers of PRWs reveals that there was only one significant difference between the 2 groups demonstrating that the attitude toward PRWs has a stronger influence on adoption of a mobile physician-rating app and on willingness to pay for it, if the respondent had no experience with PRWs in the past. All other differences in the path coefficients were not significant.

**Table 6:** Model results including group comparisons of users and nonusers of physician-rating websites (PRWs).

Hypothesis	Path description^a^	Users of PRW (n=254)		Nonusers of PRWs (n=689)		Differences (permutation test)	
		B	*P* (1 sided)	B	*P* (1 sided)	B	*P* (2 sided)
H1a	PEOU → adoption (+)	0.03	.24	0.11	<.001	–0.08	.19
H1b	PEOU → willingness to pay (+)	–0.11	.04	–0.05	.07	–0.06	.39
H2a	PU → adoption (+)	0.08	.11	–0.2	.30	0.09	.18
H2b	PU → willingness to pay (+)	0.08	.11	–0.01	.38	0.09	.22
H3a	Age → adoption (-)	–0.17	<.001	–0.07	.02	–0.10	.13
H3b	Age → willingness to pay (-)	–0.12	.01	–0.04	.10	–0.08	.23
H4a	Gender → adoption (-)	–0.02	.36	–0.08	.01	0.06	.35
H4b	Gender → willingness to pay (-)	–0.07	.14	–0.03	.17	–0.03	.62
H5a	Digital literacy → adoption (+)	0.10	.07	0.05	.10	0.05	.47
H5b	Digital literacy → willingness to pay (+)	0.03	.33	–0.03	.23	0.06	.42
H6a	Feelings → adoption (+)	0.21	<.001	0.17	<.001	0.04	.61
H6b	Feelings → willingness to pay (+)	0.09	.13	0.09	.01	0.00	.99
H7a	Patients’ value of health-related knowledgeability → adoption (+)	0.08	.09	0.06	.06	0.02	.81
H7b	Patients’ value of health-related knowledgeability → willingness to pay (+)	0.22	<.001	0.09	.02	0.13	.11
H8a	Internet use → adoption (-)	0.01	.46	–0.01	.34	0.02	.76
H8b	Internet use → willingness to pay (-)	0.02	.39	0.02	.27	0.00	.97
H9a	Internet use health → adoption (-)	–0.06	.08	–0.04	.07	–0.02	.67
H9b	Internet use health → willingness to pay (-)	0.05	.28	0.03	.15	0.02	.81
H10a	Apps use → adoption (+)	0.07	.09	0.17	<.001	–0.10	.12
H10b	Apps use → willingness to pay (+)	0.18	.01	0.24	<.001	–0.06	.49
H11a	Attitude PRWs → adoption (+)	0.27	<.001	0.43	<.001	–0.16	.03
H11b	Attitude PRWs → willingness to pay (+)	0.15	.01	0.32	<.001	–0.17	.04

^a^ PEOU: perceived ease of use; PU: perceived usefulness. A plus sign or minus sign signifies an increase or decrease, respectively, in the dependent variable evoked by an increase in the independent variable (ceteris paribus).

## Discussion

### Principal Findings

The result of the current study and the empirical testing of the conceptual model using structural equation modeling yield some interesting results. First, the results indicate that the most important factors that predict adoption of physician-rating apps and willingness to pay for physician-rating apps are present attitudes toward PRWs in general as well as frequency of apps use for health-related information in the past. Hence, if individuals have a positive attitude toward PRWs, then they are also open to apps that enable mobile access to these PRWs, and they might even be willing to pay for such apps. Similarly, if individuals already make use of other health-related apps, then they are prone to make use of physician-rating apps too, and would even accept to be charged for them.

Secondly, according to TAM, it was hypothesized that perceived usefulness of the Internet to gain health-related information and PEOU of the Internet to gain health-related information exert an influence on adoption of physician-rating apps and on willingness to pay for physician-rating apps. As expected, PEOU had a significant positive impact on adoption of physician-rating apps, but a negative impact on willingness to pay for it, in contrast to our expectations. This means that ascribing higher PEOU to the Internet to gain health-related information leads to a higher willingness to adopt physician-rating apps, but to a diminished willingness to pay for them. A possible explanation for these findings might be that there is a trade-off between Internet accessed via laptop or personal computer and Internet accessed via smartphones or tablet computers. If someone judges the Internet to be easy to use for gaining health-related information, he/she may not be willing to pay extra for the same information delivered by another sort of technological device. Additionally, these findings might also be related to the fact that a higher amount of daily private Internet use for health-related information exerted a significant negative impact on adoption of a mobile physician-rating app (see H9a). Therefore, if someone is under time pressure he/she may find it more attractive to use a PRW app instead of looking at a PRW through Internet accessed via laptop or personal computer. On the other hand, someone who currently spends more time with convenient Internet access may be less interested in a physician-rating app because the quick and ubiquitous availability, which is offered only by the mobile solution of PRW usage, may be less important for him/her.

The path coefficients from perceived usefulness to both endogenous variables were not significant (H2a+b). The results indicate that when it comes to adoption of mobile physician-rating apps, PEOU of the Internet to gain health-related information search plays a more important role than perceived usefulness of the Internet to gain health-related information. Apps are typically designed for convenient use; hence, higher PEOU of the Internet to gain health-related information leads to higher propensity to adopt physician-rating apps.

Furthermore, in-line with prior studies on the influence of age and gender on the use of the Internet concerning health-related information, the results of our study demonstrate that younger patients are more willing to adopt physician-rating apps and to pay for them. Male patients were also more willing to pay for physician-rating apps, but these differences did not meet criteria for significance.

In addition, both digital literacy and positive (affective) feelings toward the Internet proved to exert influence on physician-rating apps adoption. As was expected, individuals with higher digital literacy may see more advantages with applying the new technology of apps for PRWs and therefore are more prone to use them. However, with regard to willingness to pay for the physician-rating apps, only positive feelings toward the Internet exerts a positive influence, whereas digital literacy does not.

Another interesting finding of the current study is that patients’ value of health-related knowledgeability has a positive impact on adoption and willingness to pay. Physician-rating apps are probably perceived as devices that enable individuals to increase their knowledgeability about the physician. If patients think that being well informed is important to strengthen the communicative dimension of the relationship with the physician, mobile access to physician-related information via apps is appreciated. Interestingly, the influence of patients’ knowledgeability on willingness to pay for physician-rating apps is even stronger.

The current study reveals that the amount of daily private Internet use in general does not predict adoption and willingness to pay for physician-rating apps. The amount of daily private Internet use for health-related information search proved to be a significant predictor for the adoption of mobile physician-rating apps, but not for willingness to pay. It may be assumed that people who spend a lot of time on the Internet for health-related information searches are less interested in fast access to PRWs via apps because they may perceive a less considerable time pressure. Apps are typically designed to allow for fast access so that less time would be needed for information searching and people under time pressure are more inclined to appreciate them. But time pressure may not necessarily lead to higher willingness to pay because PRWs are accessible via normal distribution channels (laptop, personal computer) without extra costs.

Finally, group comparisons between users and nonusers of PRWs demonstrate that there is only one moderating effect of PRW usage on one of the relationships. The influence of attitude toward PRWs on adoption of mobile physician-rating apps and willingness to pay for them is moderated by usage of PRWs. Among the group of nonusers, attitude toward PRWs has a higher influence on the 2 variables than among the group of users. This may be explained by the fact that users normally have a more positive attitude toward PRWs and a smaller variability in attitude than nonusers because of their experiential background. Therefore, the predictive power of attitude toward PRWs may be lower for adoption of physician-rating apps and willingness to pay for them in the group of users than in the group of nonusers.

### Limitations and Directions for Future Research

Some limitations to the study should be noted. There is the possibility of selection bias among respondents, although random selection out of the database is held to minimize its likelihood. The recruitment rate of 64% for this online panel sample also indicates that selection bias among respondents is probably low. A demographic comparison of our sample showed that there were slightly more men and older people as well as more higher educated respondents in the sample than in the average online population of Germany. Although the number of respondents was quite high, a larger randomized sample of the average online population would be desirable.

There are also limitations concerning the survey instrument. We asked about the intended use of a hypothetical mobile physician-rating app rather than use of an existing mobile physician-rating app. Asking for an existing mobile physician-rating app was not an option for us because usage of existing mobile physician-rating apps has been scarce; therefore, only a small number of people would have been able to answer our questions. In addition, by describing a physician-rating app and asking participants to imagine it, we avoided asking participants about one specific physician-rating app because such apps differ in their quality and distribution. To draw conclusions from hypothetical variables (eg, buying intentions) to real variables (buying of products) is a common phenomenon in many social sciences. Nevertheless, some uncertainty remains about transferring the results to existing mobile physician-rating apps. Future studies might focus on existing physician-rating apps once more of these apps are available and used to a larger extent.

### Conclusion and Practical Implications

This paper analyzes the use of PRWs through mobile apps in the future. More specifically, the study identifies antecedents of physician-rating apps adoption and of willingness to pay for such apps. A mobile physician-rating app allows for flexible access to PRWs, irrespective of the individual’s location, and it may also be useful in certain circumstances (eg, when a patient is on a journey or a physician’s practice is unexpectedly closed). Several sociodemographic, psychographic, and behavioral variables of Internet use contribute to the adoption of mobile physician-rating apps and willingness to pay for mobile physician-rating apps. With regard to sociodemographic variables, male and younger patients are more prone to adopt physician-rating apps. Therefore, these target groups of mobile physician-rating apps could be used as testimonials and promoters. The first step of enhancing awareness and adoption of physician-rating apps could be to promote the physician-rating apps through social media (eg, Facebook) and other Web-based communication channels that are often used by male and younger patients. Some psychographic variables (eg, digital literacy, feelings about the Internet and Web-based applications, and value of health-related knowledgeability) support proneness of future physician-rating apps adoption. Therefore, the communication concepts for the promotors and testimonials of physician-rating apps could be tailored more specifically. In a second step of innovation diffusion of physician-rating apps the results of this study are additionally useful for creators of mobile physician-rating apps and of PRWs in general. The results have shown that the PEOU of the Internet of health-related information is a valuable antecedent of physician-rating apps adoption. Therefore, the design of physician-rating apps as well as the accessibility, usability, and user-generated content should meet the users’ requirements for further usage of physician-rating apps. The search functions should be kept simple for people who look for a physician via mobile physician-rating apps (eg, on smartphones) [88,89]. The results of this paper also reveal that an improvement of the attitude toward PRWs is also likely to lead to increased mobile physician-rating apps adoption; hence, enhancing trust in PRWs and increasing the usefulness of PRWs are critical factors for mobile physician-rating apps adoption. It might also be assumed that usage of physician-rating apps could boost the usage of PRWs in general so that PRWs could ultimately be more interesting for the populace. Additionally, higher awareness of PRWs would also lead to an even greater number of ratings per physician and the representativeness of PRWs could be enhanced. Therefore, the (nonmobile) usage of PRWs and physician-rating apps adoption are interdependent and are likely to benefit from each other.

## Multimedia Appendix 1

Construct measures used in the final PLS model and sociodemographic measures of the study.

## Abbreviations

AVE: average variance extracted
CR: composite reliability
PEOU: perceived ease of use
PLS: partial least squares
PRW: physician-rating website
TAM: Technology Acceptance Model
