# Supplementary material for: What Explains Usage of Mobile Physician-Rating Apps? Results From a Web-Based Questionnaire
Source: J Med Internet Res. 2014 Jun 11;16(6):e148. doi: 10.2196/jmir.3122 (PMC4071227; doi:10.2196/jmir.3122)
Supplement: Supplementary file 1 [file jmir_v16i6e148_app1.pdf]

ALLGEMEINE EINSTELLUNG GEGENÜBER DEM INTERNET / GENERAL ATTITUDE TOWARD THE INTERNET

1. Welche Gefühle haben Sie gegenüber dem Internet und anderen webbasierten Anwendungen (z.B. Anwendungen am Smartphone oder Tablet-PC) im Allgemeinen?

In general, what kind of feelings do you have towards the Internet and other web-based applications (e.g. apps on the smart phone or tablet)? (F1_1)

Very negative						Very positive	No answer
1	2	3	4	5	6	7	8

DIGITAL LITERACY

2. Wie würden Sie sich auf einer Skala von 1 (Ich kenne mich überhaupt nicht aus) bis 7 (Ich kenne mich sehr gut aus), im Umgang mit dem Internet im Allgemeinen, selbst einstufen?

How would you rate yourself in the handling of the Internet on a scale from 1 (I am not literate at all) to 7 (I am very literate)? (F2_1)

Not literate at all						Very literate	No answer
1	2	3	4	5	6	7	8

NUTZUNGSVERHALTEN / INFORMATION SEEKING BEHAVIOR

3. Wenn Sie an Ihre Internetnutzung denken, wie viele Stunden nutzen Sie durchschnittlich das Internet für private Tätigkeiten? (Bitte wählen Sie eine Antwortoption (pro Tag oder pro Woche oder pro Monat) aus, die Ihrer Nutzung am ehesten entspricht)

Durchschnittliche Internetnutzung in **Stunden pro Tag** __________

Durchschnittliche Internetnutzung in **Stunden pro Woche** __________

Durchschnittliche Internetnutzung in **Stunden pro Monat** __________

If you think about your Internet usage, how many hours do you use the Internet for private tasks on average? (Please select one answer option (per day or per week or per month), which corresponds to your usage most likely) (F3)

Average Internet usage **in hours per day** __________

Average Internet usage **in hours per week** __________

Average Internet usage **in hours per month** __________

4. Wie viele Stunden nutzen Sie durchschnittlich das Internet für die Suche nach gesundheitsorientierten Informationen?

(Bitte wählen Sie eine Antwortoption (pro Tag oder pro Woche oder pro Monat) aus, die Ihrer Nutzung am ehesten entspricht)

Durchschnittliche Internetnutzung in **Stunden pro Tag** __________

Durchschnittliche Internetnutzung in **Stunden pro Woche** __________

Durchschnittliche Internetnutzung in **Stunden pro Monat** __________

How many hours on average do you use the Internet for searching for health related information? (F4)

Average Internet usage **in hours per day** ___________

Average Internet usage **in hours per week** ___________

Average Internet usage **in hours per month** ___________

5. Bitte kreuzen Sie an, wie häufig Sie die folgenden Kanäle im Internet für die Suche nach gesundheitsorientierten Informationen nutzen.

		Erläuterung	Täglich	Wöchentlich	Seltener als wöchentlich	Monatlich	Seltener als monatlich	Nie
F7_10	Apps	*Eine Applikation (kurz: App) ist eine Anwendung auf Smartphones – vergleichbar mit einem Programm am Computer.*	1	2	3	4	5	6

Please mark how often you use the following channels on the Internet for health related information searches.

		Annotation	Daily	Weekly	Less often than weekly	Monthly	Less often than monthly	Never
F7_10	Apps	*An application (briefly: app) is a task on smartphones – comparable to a software program on a computer.*	1	2	3	4	5	6

6. Bitte kreuzen Sie an, aus welchen Gründen Sie das Internet für die Suche nach gesundheitsorientierten Informationen nutzen?

	**Ich nutze das Internet, weil …**	Trifft überhaupt nicht zu						Trifft vollkommen zu	Keine Antwort
F11_1	… ich auf einfache Art und Weise Recherchen machen kann.	1	2	3	4	5	6	7	8
F11_2	… es die Informationssuche für mich erleichtert.	1	2	3	4	5	6	7	8
F11_11	… ich auf einfache Art und Weise mit jemandem in Kontakt treten kann.	1	2	3	4	5	6	7	8
F11_12	… ich am Puls der Zeit sein möchte.	1	2	3	4	5	6	7	8
F11_15	… ich mein Wissen mit anderen teilen kann.	1	2	3	4	5	6	7	8

For which reasons do you use the Internet for health related information searches? Please mark all that apply.

	**I use the Internet, because…**	Strongly disagree						Strongly agree	No answer
F11_1	… I can search online easily.	1	2	3	4	5	6	7	8
F11_2	… it facilitates the information search for me .	1	2	3	4	5	6	7	8
F11_11	… I can make contact with someone easily.	1	2	3	4	5	6	7	8
F11_12	… I want to be up-to-date.	1	2	3	4	5	6	7	8
F11_15	… I can share my knowhow with others.	1	2	3	4	5	6	7	8

INTERNETKOMMUNIKATION MIT DEM ARZT / INTERNET COMMUNICATION WITH THE PHYSICIAN

7. Bitte geben Sie im Folgenden an, wie sehr die einzelnen Aussagen auf Sie zutreffen.

		Trifft überhaupt nicht auf mich zu						Trifft sehr auf mich zu	Keine Antwort
F20_5	Ist der Patient/die Patientin informiert, wird die Kommunikation mit dem Arzt/der Ärztin dadurch verbessert.	1	2	3	4	5	6	7	8
F20_8	Ist der Patient/die Patientin informiert, nimmt sich der Arzt/die Ärztin mehr Zeit für die Behandlung.	1	2	3	4	5	6	7	8
F20_9	Der Arzt/die Ärztin verschreibt eher ein gewünschtes Medikament, wenn der Patient/die Patientin informiert ist.	1	2	3	4	5	6	7	8

Please indicate how much the following statements apply to you.

		Strongly disagree						Strongly agree	No answer
F20_5	If the patient is informed, the communication with the physician will be improved.	1	2	3	4	5	6	7	8
F20_8	If the patient is informed, the physician takes more time for the treatment.	1	2	3	4	5	6	7	8
F20_9	The physician prescribes a medicine more probably, if the patient is informed.	1	2	3	4	5	6	7	8

REZENSIONEN / REVIEWS

8. Haben Sie schon einmal Informationen über einen Arzt / eine Ärztin auf einer solchen Plattform eingeholt?

1 Ja 2 Nein 3 Keine Antwort

Have you ever gathered information on a physician at a PRW? (F22)

1 Yes 2 No 3 No answer

9. Wie wahrscheinlich ist es, dass Sie in Zukunft eine solche Empfehlungsplattform für Ärzte/Ärztinnen nutzen werden?

Überhaupt nicht wahrscheinlich						Sehr wahrscheinlich	Keine Antwort
1	2	3	4	5	6	7	8

How probable is it that you will use PRWs in the future? (F25_1)

Not probable at all						Very probable	No answer
1	2	3	4	5	6	7	8

10. Wie nützlich finden Sie solche Empfehlungsplattformen für die Arztsuche im Vergleich zu anderen Empfehlungsquellen (z.B. andere Ärzte, Familie, Bekannte etc.)?

Überhaupt nicht nützlich						Sehr nützlich	Keine Antwort
1	2	3	4	5	6	7	8

How useful are PRWs in comparison to other recommendation sources (e.g. other physicians, family, friends etc.) from your point of view? (F26_1)

Not at all useful						Very useful	No answer
1	2	3	4	5	6	7	8

11. Inwieweit vertrauen Sie den Informationen auf Empfehlungsplattformen für Ärzte und Ärztinnen?

Ich habe überhaupt kein Vertrauen.						Ich habe sehr großes Vertrauen.	Keine Antwort
1	2	3	4	5	6	7	8

How much do you trust the information provided on PRWs? (F27_1)

No trust at all						Very high trust	No answer
1	2	3	4	5	6	7	8

12. Im Folgenden handeln die Fragen von mobilen Geräten, im Speziellen von Smartphones und Applikationen. Bitte beantworten Sie die Statements nach dem Einleitungstext.

Stellen Sie sich vor, es gäbe für Smartphones ein App (eine Applikation) zur einfachen Suche nach ÄrztInnen. Dem User bzw. der Userin wäre es möglich, in ein Suchfeld ein Symptom, an dem er/sie leidet einzugeben und als Ergebnis käme eine Auflistung mit allen in Frage kommenden ÄrztInnen in der unmittelbaren Umgebung, inklusive Bewertungen von anderen PatientInnen hinsichtlich Zufriedenheit mit dem Arzt/der Ärztin, Atmosphäre der Praxis, Wartezeiten, Behandlung etc.

		Trifft überhaupt nicht zu						Trifft voll-kommen zu	Keine Antwort
F28_1	Ich finde ein solches App gut.	1	2	3	4	5	6	7	8
F28_2	Ich kann mir vorstellen dieses App zu nutzen.	1	2	3	4	5	6	7	8
F28_3	Ich würde für dieses App zahlen.	1	2	3	4	5	6	7	8

The following questions are about mobile devices, particularly about smartphones and applications. Please answer the statements after reading the introduction section.

Imagine there is an app for smartphones that allows you to search for physicians. The user could fill in a symptom of a condition and as a result, all physicians in the surrounding area would be listed, including the ratings of these physicians according to the rating patients' satisfaction with him/her, with the atmosphere of the waiting room, waiting time, the treatment, et cetera.

		Strongly disagree						Strongly agree	No answer
F28_1	I appreciate such an app.	1	2	3	4	5	6	7	8
F28_2	I am willing to use such an app.	1	2	3	4	5	6	7	8
F28_3	I am willing to pay for such an app.	1	2	3	4	5	6	7	8

DEMOGRAPHISCHE ANGABEN / SOCIODEMOGRAPHIC DATA

D1 Geschlecht: [1] männlich
[2] weiblich

D2_1 Geburtsjahr: _______________

D1 Gender: [1] male
[2] female

D2_1 Year of birth: _______________

Höchste abgeschlossene Ausbildung:

1 Schüler in allgemeinbildender Schule (ohne Schulabschluss)
2 Haupt-/Volksschulabschluss ohne Lehre
3 Haupt-/Volksschulabschluss mit Lehre
4 Weiterführende Schule ohne Abitur (Realschulabschluss / Mittlere Reife)
5 Abitur / (Fach-) Hochschulreife ohne Studium
6 Abitur / (Fach-) Hochschulreife mit Studium
7 Promotion/ Habilitation
8 Keine Antwort

Highest level of education: (D4)

1 Without school qualification
2 Secondary general school
3 Polytechnic secondary school

4 Intermediate secondary school
5 High school diploma / A-levels
6 University degree
7 Postdoctoral degree / Professor
8 No answer

Familienstand:

1 Ledig
2 In einer Partnerschaft
3 Verheiratet
4 Geschieden
5 Verwitwet
6 Keine Antwort

Marital status: (D5)

1 Single
2 Close-partnered
3 Married
4 Divorced
5 Widowed
6 No answer

D6_1 **Anzahl der Personen im Haushalt: ___________**

D6_1 **Number of individuals in the household: ___________**
